# Quantification of Ceramsite Granules in Lightweight Concrete Panels through an Image Analysis Technique

**DOI:** 10.3390/ma15031063

**Published:** 2022-01-29

**Authors:** Changming Bu, Haiyan Yang, Lei Liu, Dongxu Zhu, Yi Sun, Linwen Yu, Yuhui Ouyang, Xuemei Cao, Qike Wei

**Affiliations:** 1 School of Civil Engineering and Architecture, Chongqing University of Science & Technology, Chongqing 401331, China; buchangming@cqust.edu.cn (C.B.); 2019206041@cqust.edu.cn (H.Y.); 2020206111@cqust.edu.cn (L.L.); 2020206029@cqust.edu.cn (D.Z.); 2020206038@cqust.edu.cn (Y.O.); 2020206051@cqust.edu.cn (X.C.); 2 Chongqing Key Laboratory of Energy Engineering Mechanics & Disaster Prevention and Mitigation, Chongqing 401331, China; 3 College of Materials Science and Engineering, Chongqing University, Chongqing 400044, China; linwen.yu@cqu.edu.cn; 4 China Metallurgical Construction Engineering Group Construction, Ltd., Chongqing 400084, China; weiqike@cmccltd.com

**Keywords:** ceramsite concrete, lightweight concrete wall panels, density, compressive strength, ultrasonic pulse velocity, quantitative study

## Abstract

Ceramsite particles are an important component of lightweight ceramsite concrete wall panels, and the density of the aggregate is much lower than the density of the slurry. It is generally accepted that there are inhomogeneities in the distribution of ceramsite particles in wall panels. Ceramsite concrete wallboard material is a research hotspot in the field of fabricated building materials at home and abroad; however, there is no effective way to quantify their inhomogeneity. Based on the application of image recognition technology in concrete homogeneity, a method to quantitatively evaluate the distribution of light aggregates in wall panels was developed. Three commercial lightweight vitrified concrete wall panels were cut into 324 cubes. The four cut surfaces of each specimen were photographed to analyze the proportion of ceramsite particle area, while the density, ultrasonic pulse velocity, and compressive strength of the specimens were tested. The results demonstrated that the image analysis method could effectively describe the homogeneity of the panels. The proportion of particle area of aggregate in the section of the cube had a strong correlation with the compressive strength, ultrasonic pulse velocity, and density, and there was an obvious linear relationship with the height of the plate where the cube was located. Based on this, the correlation equations of the proportion of particle area of aggregate, density, ultrasonic pulse velocity, compressive strength, and the height where the specimen was located were proposed. The quantitative parameters of the relevant properties of the wall panels were also obtained: the maximum difference between the proportion of particle area of the aggregate was 24%, the maximum difference between the density at the top and bottom of the wall panels was 115 kg/m^3^, and the maximum difference in the strength reached 5 MPa.

## 1. Introduction

Lightweight concrete wall panels are mostly used as non-load-bearing structural elements in assembled buildings, usually as partition walls [1] or as composite panels with other materials for exterior walls [2]. As an assembled building, being lightweight is the primary requirement [3], and a lighter wall panel weight can help reduce the overall weight of the assembled building while reducing the permanent load of the load-bearing panels in the structure. Ceramsite concrete wall panels have become mainstream in the fabricated building wallboard market due to their low heat conductivity [4], low density [5], good permeability resistance [6], low early age autogenous shrinkage [7], and early and faster gains in strength [8].

However, as a lightweight concrete wallboard, its strength index also needs to be fully considered. Some studies have summarized the relevant laws to draw general conclusions that the strength of lightweight concrete materials increases with increasing density [9], with a different emphasis for different structural parts [10], such as the compressive strength of compression members and the flexural strength of tension bending members. Although normal-weight aggregate concrete performed better, lightweight aggregate concrete weighed less and had a positive effect on the environment [11], lower early age autogenous shrinkage [12], relatively good thermal insulation performance [13], less detrimental impacts of solid waste on the environment, and also addressed the shortage of natural resources [14]. Some scholars have discussed the influence of substitution rate [8], shape [15], materials [16], and type [17] of the ceramsite, silica fume [18], and CNTs [19] on the compressive strength of lightweight aggregate concrete. 

Some scholars have shown that there is a significant density difference between ceramsite and cement paste [20], the antisegregation of lightweight aggregate is weak, and the segregation phenomenon of ceramsite concrete is more significant [21], so the aggregate cannot be evenly distributed in the concrete, thus affecting the strength of the specimens [22]. Compared with ordinary concrete, one of the biggest disadvantages of lightweight concrete is the unevenness of aggregate in the mixture. The uneven distribution of aggregate will seriously affect the working performance of concrete materials and have a negative impact. In order to reduce the segregation phenomenon [23,24], most engineers adopt a way of optimizing the means of vibrating and reducing the time of vibrating [25,26], which requires the construction personnel to finish a large degree of slurry vibration in a short time [21]. In addition, the goal of reducing the segregation phenomenon can also be achieved by modifying the ceramsite granules. Most often, prewetted ceramsite granules are used to reduce the difference in density [27], adjust the ceramsite granule gradation [28], and reduce the aggregate particle size [29]. Increasing the surface roughness of the aggregate can also help to reduce the segregation phenomenon [30]. In recent years, image recognition technology has become an effective technique for identifying the surface and internal structure of structures and is widely used for rapid crack detection, damage monitoring [31], and safety assessment of bridge structures, highways [32], and subway tunnels [33]. Image recognition techniques can also be used for the detection of homogeneity in vitrified concrete wall panels and can help to determine the distribution of vitrified particles in strong panel material [34]. Although there are many methods to study the unevenness of concrete, applying these methods to the quantification of commercial ceramsite concrete wallboard is still lacking. 

Based on the actual engineering needs, this paper quantifies the uneven distribution of ceramsite in wallboard by using the uniformity evaluation method. The wallboard was cut into cubes and its physical and mechanical properties were tested. In addition, the distribution of ceramsite was studied by image analysis technology. The main purpose was to reveal and quantify the uneven distribution of ceramsite concrete wallboard and provide reference data for its application in engineering.

## 2. Experimental Details

### 2.1. Geometry of the Wall Panels

**Figure 1:**
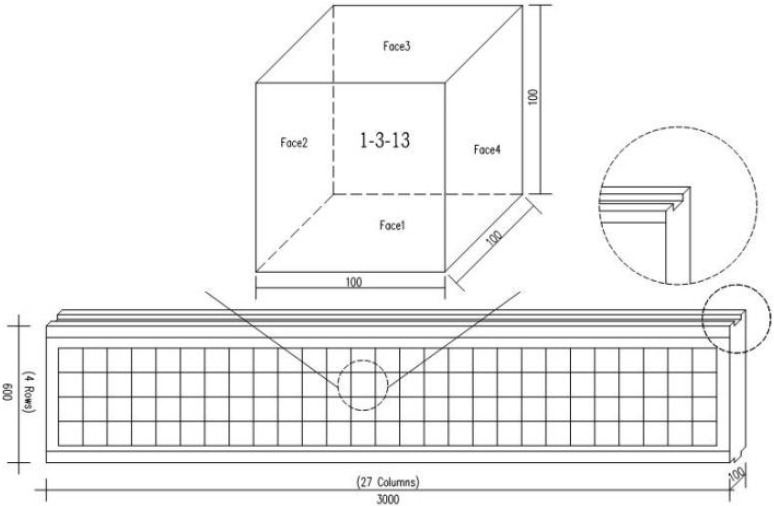
Schematic diagram of wall panel cuttings.

The size of the wall panels was 3000 × 600 (width × height, in mm), and their thicknesses were 90 mm, 100 mm, 120 mm, and 150 mm. The thickness of the wall panels selected for this study was 100 mm. The schematic diagram of the wall panel cutting is shown in Figure 1.

### 2.2. Material Composition of Wall Panels 

The wall panels used in the present study were developed by Chongqing Chengwei Lightweight Wallboard Co., Ltd. The mix ratio shown in Table 1 was also provided by the company, and was obtained many times by the factory for research, development, and improvement. The water-cement ratio was 0.4, the aggregate of the wallboard used in this paper was clay ceramsite, and the relevant parameters were as follows: the particle size range was 0–10 mm, the density grade was 500, the bulk density was 476 kg/m^3^, and the moisture content was 16.63%. The chemical composition of the clay used to make ceramsite included quartz, montmorillonite, chlorite, etc. The materials and mix ratios used in the slab are shown in Table 1.The slump value of the freshly poured concrete complied with Chinese standard GB/T50080-2016. Fresh concrete was mixed well and poured into each formwork below from above, and due to the presence of light aggregates, a small action of plugging and pounding was carried out to avoid serious delamination and segregation.

### 2.3. Test Procedures

**Figure 2:**
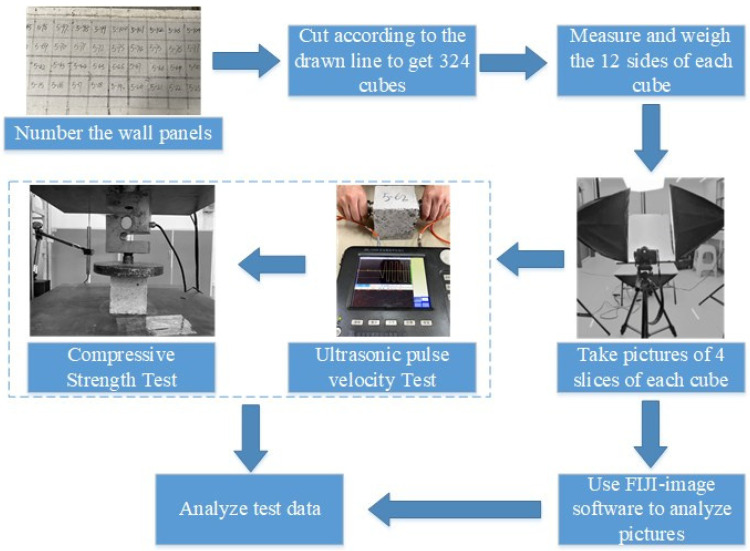
Test procedures.

The test procedures followed in this paper are shown in Figure 2.

#### 2.3.1. Preparation of Specimens

To investigate the inhomogeneous distribution of ceramsite particles in commercial ceramsite concrete wall panels, three 100 mm thick commercial ceramsite concrete wall panels were cut into 100 mm × 100 mm × 100 mm cubic specimens, and all three wall panels were hardened under natural curing conditions. The upper part of the wall panels had a groove for connection, and the lower part had a projection for connection. The upper and lower parts of the wall panels had 100 mm in an inclined state, therefore, the above factors were excluded when cutting the wallboards. At the same time, during the cutting process, the thickness of the cutting blade caused some wear and tear. Finally, each wallboard was cut into 4 rows and 27 columns of samples and marked as A-B-CC, with A indicating the number of the wallboard (1 digit), B indicating the row number (1 digit), and CC indicating the column number (2 digits), as shown in Figure 1.

The method of cutting the wall panels was important for the image identification results; to reduce the effect of specimen cutting on the image identification results, no water was poured during the cutting. Because of the certain strength, this test used a cutting table for the preparation of specimens. In specimen preparation, due to the manual cutting of workers and the strength of the specimen, some specimens had certain cutting scratches on the surface, which was unavoidable.

#### 2.3.2. Photography and Analysis

**Figure 3:**
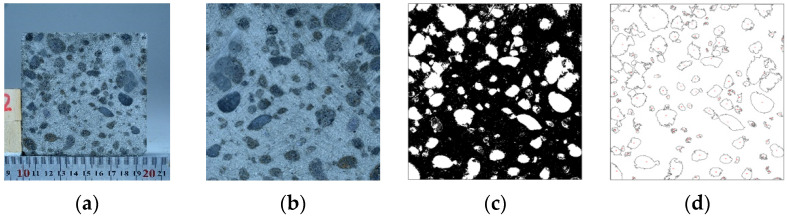
Image analysis process: (**a**) original photo; (**b**) cropped photo; (**c**) after binarization; (**d**) outlines of ceramsite beads.

In each cubic specimen, there were four faces that showed the distribution of ceramsite particles in the concrete. The photos of each face were taken from the direction perpendicular to the face. In order to reduce the error in the image recognition analysis, the position of the camera (SONY ILCE-7RM3 includes FE 24-70mm F2.8 GM, Japan, Tokyo) and the placement table was fixed in the photograph of the specimen, and the photograph of each face was taken from the direction perpendicular to the face. Therefore, the distance between the photographic surface and the camera was the same for all specimens, and the actual length of the specimen matched the number of pixels in the graph. Fiji-ImageJ (ImageJ-win64)software was used to complete the recognition of image particles; the main steps of the image recognition are shown below and in Figure 3.

1. The standard scale of the image was used to obtain the relationship between the pixels and the actual size of the image.
2. The rectangular area at the edge was cropped off by Photoshop (PS, Adobe Photoshop CC 2019) to make sure that only the part of vitrified concrete was left in the picture.
3. To distinguish the cement paste from the ceramsite particles, the colored pictures were changed to gray, different algorithms were analyzed and compared, and the automatic global threshold processing method was considered most appropriate.
4. Using the image analysis software program ImageJ, the area of surface ceramsite particles was calculated.

#### 2.3.3. Density, Ultrasonic Pulse Velocity and Compressive Strength Test

The density of ceramsite concrete was obtained by measuring the actual length of 12 sides of each specimen with vernier calipers, accurate to 1 mm, and weighing all cubic specimens in their natural environment.

A nonmetallic ultrasonic detector (ZBL-U520, produced by Beijing Zhibolian Technology Co., Ltd., Beijing, China) was used to test the ultrasonic pulse velocity. Two transducers were aligned to both sides of the specimen, respectively, the ultrasonic pulse wave was recorded through the specimen for a time, and the ultrasonic pulse velocity was calculated according to Equation (1).

(1) $$V=\frac{L}{T}$$

where *V* is the ultrasonic pulse velocity of the specimens (km/s), *L* is the distance of the ultrasonic wave passing through, and *T* is the time of ultrasonic wave passing (s).

Every specimen was put into a testing machine with a 300 kN capacity flatwise, keeping the two facings at the bottom and top to obtain the compressive strength of the core, and the compressive strength was obtained by a dynamic high-speed data acquisition system (DH8303), and the loading velocity was 0.2 MPa/s.

## 3. Test Results and Discussion

### 3.1. Distribution of Ceramsite Granules

Although the total amount of ceramsite particles in the concrete was determined by the mix ratio of the wall panels, the percentage of particles in each specimen was inconsistent. The three-dimensional distribution information of particles within the specimen was analyzed by two-dimensional images of the surface of the cut specimen, and when hundreds of images were used to analyze and compare this information, a quantitative distribution of particles could be achieved.

As shown in Figure 1, each cube had six faces, and the distribution of ceramsite particles can be seen on all four faces, except for the top and bottom faces of the specimen. The four faces of ceramsite concrete were photographed and analyzed using ImageJ software. The distribution of the percentage of ceramsite particles is discussed below.

#### 3.1.1. Determination of Available Image Areas

A standard ruler was placed on the photo storage table to obtain the relationship between the actual length of the photo and the pixel points of the picture, and then the information of the shape of ceramsite particles was analyzed. Due to the use of manual cutting, the boundary of the specimens could not be idealized into a straight line, and some particles at the edge had a certain impact, so these areas were excluded from the analysis.

The effects of the areas of the selected regions on the results are discussed. A picture of the cut face was selected at random, and 11 rectangular regions of different edge lengths, from 1258 × 1265 to 1258 × 1275 pixels with increments of 10 pixels, were selected for analysis. The results show that with the enlargement of the area of the selected region, the percentage of the area of the ceramsite beads to the area of the selected region was almost the same: the mean value was 37.94%, the standard deviation was 0.01%, and the coefficient of variation was only 0.03.

By selecting rectangular areas with different edge lengths, we found that with a slight change of section area, the percentage of ceramsite particles in the area remained almost unchanged. Therefore, it was reasonable to manually select the image area.

#### 3.1.2. Image Analysis Results

In this paper, the *X*-axis in the cloud chart represents the abscissa of the sample, and the *Y*-axis represents the ordinate of the sample.

**Figure 4:**
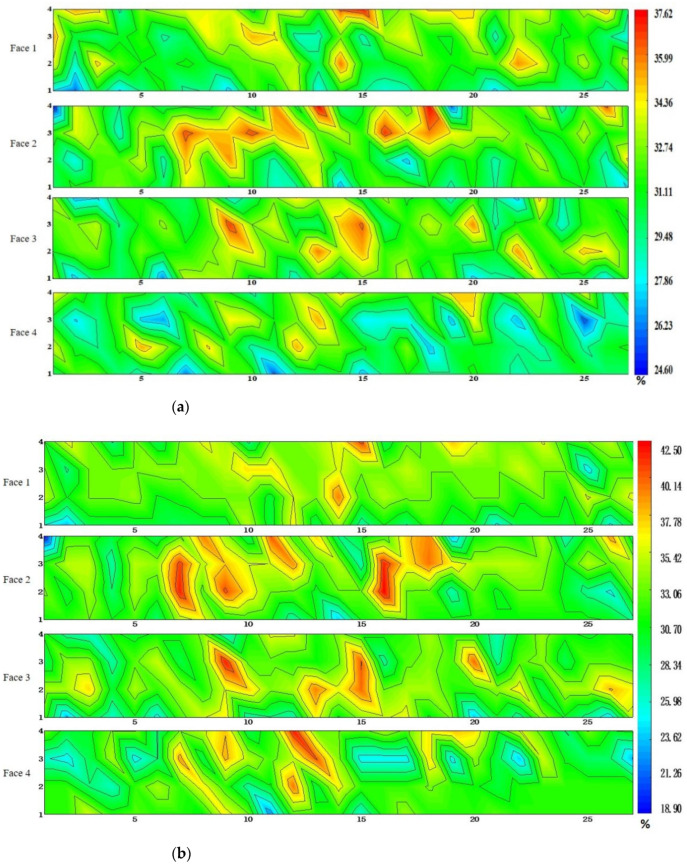

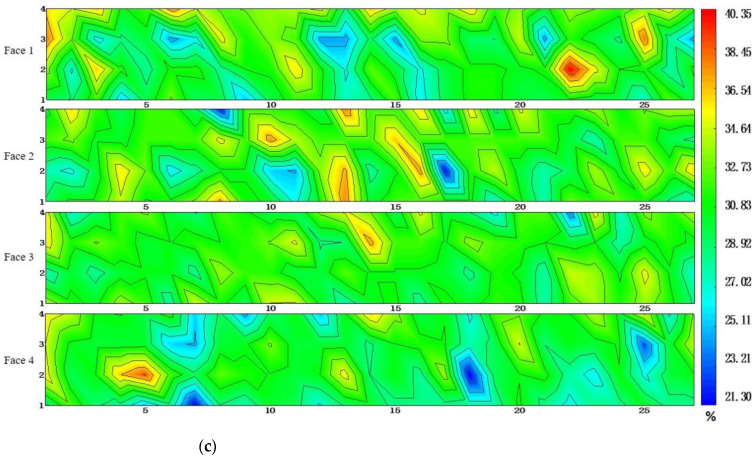
Contours of the area ratios of the ceramsite beads in the cut faces: (**a**) panel 1, (**b**) panel 2, (**c**) panel 3.

Combined with the location information of the specimens, the proportional area of ceramsite particles on each wall panel could be expressed on four sides, as shown in Figure 4. The results show that the distribution of particles on the four sides of each wall panel strongly correlated. The proportion of particles at the lower part of each side of each wall panel was lower than that at the upper part. With increased height, the proportion of ceramsite particles gradually increased, with only a small aggregate of ceramsite particles at the top of the wallboard. The minimum particle areas of the three wallboards were 24.68%, 18.9%, and 21.3%, respectively, the maximum particle areas were 37.62%, 42.5%, and 40.35%, and the differences between the maximum and minimum values were 13%, 23.6%, and 19.05%. The floating of the aggregate is caused by the density difference between the mortar and the aggregate. An aggregate with a larger particle size can obtain greater buoyancy, thus causing unevenness of the aggregate. Therefore, the image analysis method can reflect the unevenness in the concrete to a certain extent [35]. 

Faces 1 and 3 and faces 2 and 4 of the sample are opposite faces. When the thickness of the cut sample is small enough (up to dx), the particle area ratios of faces 1 and 3 and faces 2 and 4 should be consistent. In this paper, since the thickness of the specimen was 100 mm, there were some differences in the opposite sides, but the general trend remained the same. For example, in Figure 3a, when the abscissa of the sample was 10 and the ordinate was about 3, although the particle area ratios of each surface were quite different, they all reached the larger value of the surface where they were located. It can be seen that the proportions of prominent larger and smaller particle areas were consistent in faces 1 and 3 as well as faces 2 and 4 in the tangential grain area distribution of each wallboard.

At the same time, there are some abnormal situations in the figures. For example, in the part of the middle height of wallboard 2, the proportion of particles is greater than that of the surrounding particle area. This was found to be caused by the insertion and tamping process after pouring, and the insertion and tamping process of wallboard pouring were completed by a hooked reinforcement. When the wallboard is rammed with steel bars, the ceramsite in the lower part lifts, causing the ceramsite to gather in the upper part of the middle of the wallboard.

**Figure 5:**
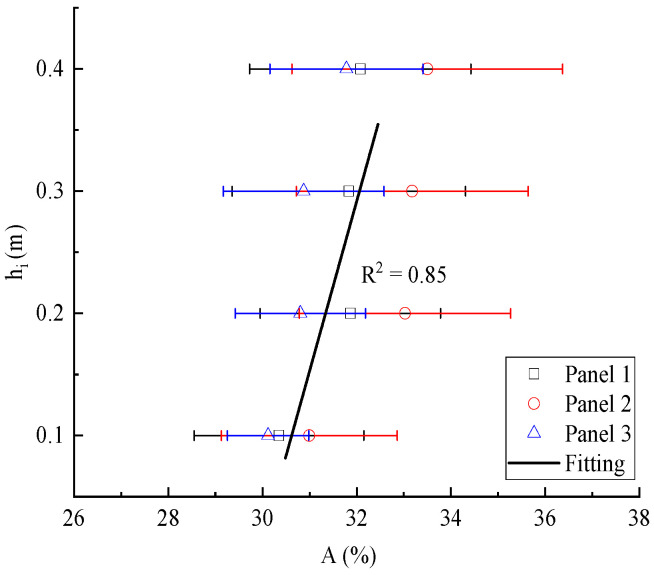
Relationship between proportion of ceramsite area and height.

In order to discuss the effect of height on the proportion of particles, the height variation trend was studied. We used an analysis of variance (ANOVA) to verify the differences among the sample test groups. After the analysis of variance, the results showed that F = 14.24, *p* = 7 × 10^−6^ < 0.05 for wall panel 1, F = 4.85, *p* = 0.04 < 0.05 for wall panel 2, F = 6.15, *p* = 6.8 × 10^−4^ < 0.05 for wall panel 3; with the change in the height of the sample from the ground, the difference in the proportion of particle area was statistically significant. Since the distribution trend of the particle area ratio of the four faces of each wall panel was similar, the average value of the particle area ratio of the four faces was obtained, and then the average value of the particle area ratio of the 27 specimens in each row of the wall panel was calculated. As shown in Figure 5, the area ratio of particles increases with the increase in height. After linear fitting, the relationship between the area ratio of particles and the height was obtained, as shown in Equation (2):

(2) 0.14A=4.15+hi

where *h_i_* is the distance from the top of the specimens to the ground and *A* is the particle area proportion of the specimens at *h_i_*.

### 3.2. Ultrasonic Pulse Velocity

Nonmetallic, ultrasonic, nondestructive testing technology can be used to analyze the filling capacity and homogeneity of the concrete. The purpose of this experimental research was to analyze the correlation between the height and the ultrasonic pulse velocity of the specimen. The ultrasonic velocity of the specimen was calculated and analyzed by testing the duration of passage of the ultrasonic pulse through the specimen.

**Figure 6:**
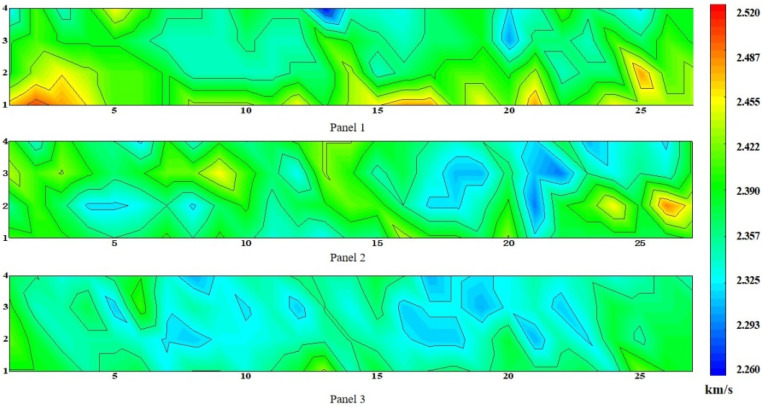
Ultrasonic pulse velocity distribution nephogram.

The ultrasonic pulse velocity distribution nephogram of the three wallboards is shown in Figure 6. Each wallboard was cut into 108 specimens, named 1–108, respectively. With the increase in height of the specimens from the ground, the ultrasonic pulse velocity of the specimens decreased gradually. Because of the increase in ceramsite particles in the specimens, the time required for the ultrasonic wave to pass through the specimens increases and the ultrasonic pulse velocity decreases. This phenomenon is consistent with the conclusion of the previous section, because there are more particles at the top, the ultrasonic velocity at the top is smaller.

It can be seen from Figure 6 that the ultrasonic pulse velocity at the bottom is basically the highest for the three wallboards. On the one hand, there is a large amount of cement paste at the bottom and the cement paste is the component that really changes the velocity [36]. The energy consumed by ultrasonic waves passing through cement paste is lower than that passing through the lightweight aggregate; therefore, the time for the ultrasonic pulse wave to pass through the former is lower than that passing through the latter. On the other hand, the agglomeration of the upper ceramsite results in a lack of tight bonding with the cement paste, resulting in excessive pores, and the wave propagation speed in the air will be much slower than that in the solid.

**Figure 7:**
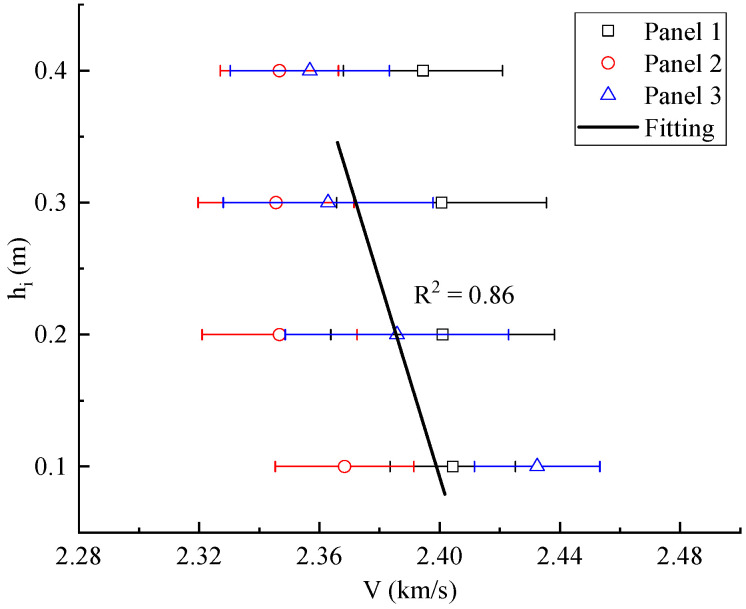
Variation trend of ultrasonic pulse velocity with height.

In order to discuss the effect of height on ultrasonic pulse velocity, the trend of its variation with height was analyzed, and its calculation method was consistent with the calculation of the correlation between the percentage of particle area and height. Through the analysis of variance, the *p* values of the sample data were all less than 0.05, and the coefficients of variation were 0.015, 0.013, and 0.01, respectively. As shown in Figure 7, with the increase in height, the ultrasonic pulse velocity of the specimens gradually decreased. The relationship between ultrasonic pulse velocity and height was obtained, as shown in Equation (3):

(3) 7.46V=18.0−hi

where *h_i_* is the distance from the top of the specimens to the ground, and *V* is the ultrasonic pulse velocity of the specimens at *h_i_*.

### 3.3. Distribution of Density and Compressive Strength

**Figure 8:**
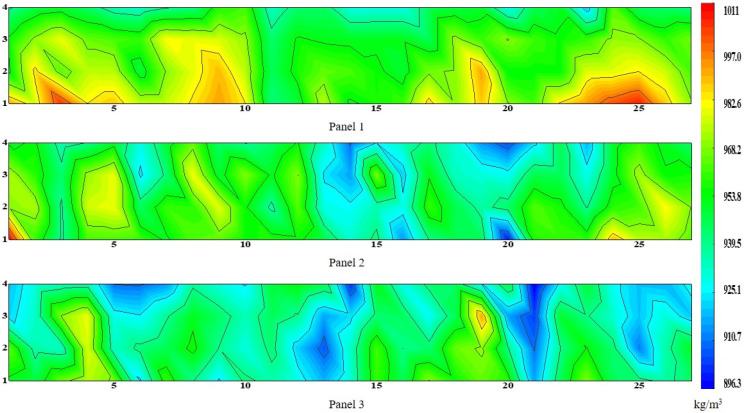
Density distribution nephogram.

**Figure 9:**
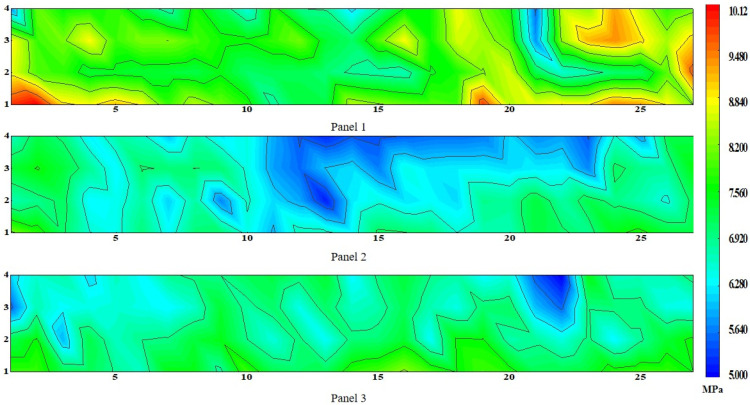
Compressive strength distribution nephogram.

Figure 8 and Figure 9 show the density distribution cloud map and compressive strength distribution cloud map of the wallboard, respectively. It can be clearly seen that the density and compressive strength of the bottom of the wall panel are larger, and the opposite is true at the top. From the bottom to the top, the density decreased from 1010 kg/m^3^ to 896 kg/m^3^, and the compressive strength decreased from 10 MPa to 5 MPa. Due to the nonuniformity of the particles, the density of the middle part of plates 2 and 3 was relatively discrete, the compressive strength and density of the specimens were inversely proportional to the proportion of particle area, the proportion of particle area increased, and the compressive strength and density of the specimens decreased. 

**Figure 10:**
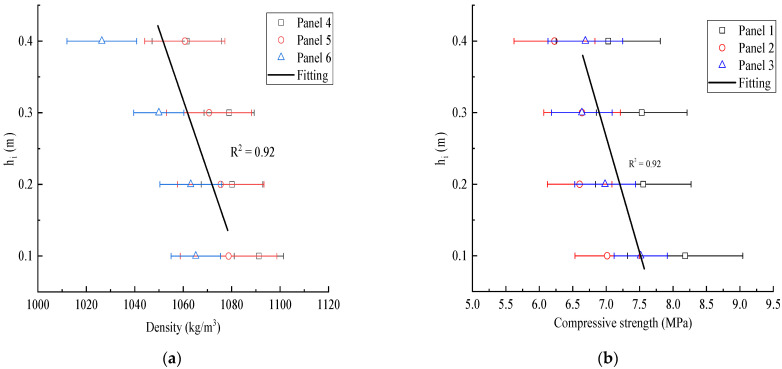
Graphs of parameter variation with height: (**a**) variation trend of density with height; (**b**) variation trend of compressive strength with height.

As shown in Figure 10, a specimen’s density and compressive strength tended to decrease with increasing height. The *Y*-axis represents the height, which is the top of the specimen from the ground, and the *X*-axis represents density and compressive strength in Figure 10a,b, respectively. By taking the average of 27 specimens in each row, the density and compressive strength of the specimens were obtained in relation to the height. As shown by the fitting curve in the figure, as the height decreased, the density and compressive strength of the specimen gradually increased. 

**Figure 11:**
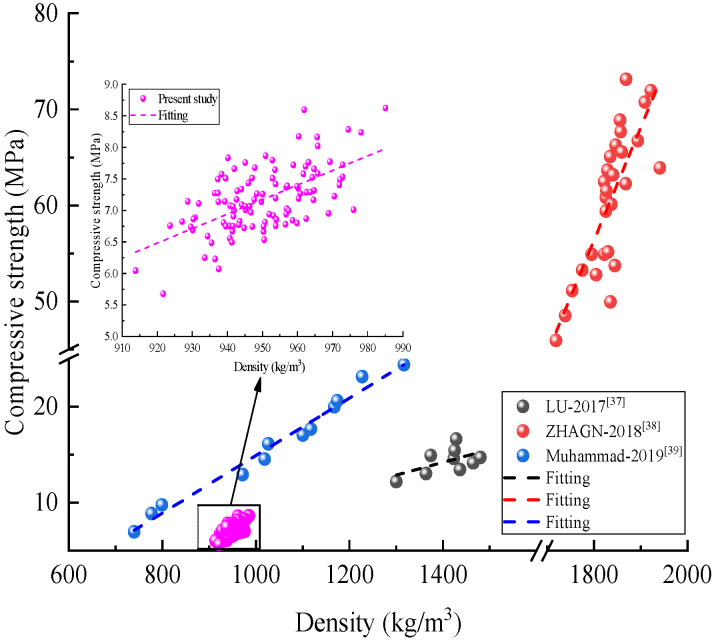
Comparison with previous studies [37,38,39].

By varying six different factors on density and compressive strength, Lu et al. [37] found a positive correlation between the changes in density and compressive strength. Meanwhile, Zhang et al. [38] suggested that the strength increases with increasing density in a basically linear relationship and Ahmad et al. [39] considered that density has a direct relationship with the strength (the higher the density, the higher the strength). Previous studies [37,38,39] have shown that the compressive strength increases as the density of the specimen increases, which is consistent with the phenomena shown in this paper (Figure 11). The decrease in specimen density with height is because there are more aggregates at the top, and at the same time the thickness of the gel between the aggregates becomes smaller, resulting in a decrease in the compression resistance of the specimen.

The average of 27 specimens in each row of each wall panel can be used to obtain the average density and height distribution of compressive strength in the corresponding row. It can be seen that these values seem to have a linear relationship with the height, and the changes in density and compressive strength with the increase in height comply with Equations (4) and (5):

(4) 0.01ρ=12.27−hi

(5) 0.32fc=2.53−hi

where *h_i_* is the distance from the top of the specimens to the ground, ρ is the density of the specimens at h_i_, and $f_{c}$ is the compressive strength of the specimens at *h_i_*_._

**Figure 12:**
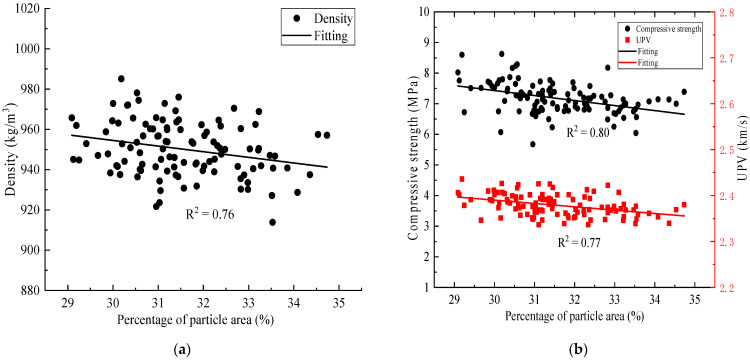
Parameter Correlation: (**a**) relationship between percentage of particle area and density; (**b**) percentage of particle area in relation to compressive strength and UPV.

Integrating the above equations, the relationship between the density, the compressive strength, the ultrasonic pulse velocity and the percentage of the particle area of the specimen can be obtained as shown in Equations (6)–(8) below. There is a certain correlation between the density, compressive strength, and ultrasonic pulse velocity of the specimens and the proportion of the specimen’s particle area. With an increase in the proportion of particle area, the density, compressive strength, and ultrasonic pulse velocity of the specimens gradually decreased, as shown in Figure 12.

(6) 0.01ρ=16.42−0.14A

(7) 7.46V=13.85−0.14A

(8) 0.32fc=6.68−0.14A

Here, *h_i_* is the distance from the top of the specimens to the ground, ρ is the density of the specimens at *h_i_*, $f_{c}$ is the compressive strength of the specimens at *h_i_*, and *V* is the ultrasonic pulse velocity of the specimens at *hi*. 

The proportion of particle area, density, and strength distribution of commercial ceramsite concrete wallboards were quantified, and the corresponding correlation formula was established, which has great reference value for the performance optimization, production, and installation of commercial ceramsite concrete wallboards. The proportion difference of ceramsite particles is due to the uneven distribution of wallboard particles, resulting in uneven distribution of particle area, density, ultrasonic velocity and compressive strength of wallboard.

## 4. Conclusions

This paper quantified the inhomogeneity of commercial ceramsite concrete wall panels through test methods such as image recognition, compressive strength, and ultrasonic velocity. We have provided relevant reference data for the production and use of wallboards that can help factories to optimize the production of wallboards. The following conclusions can be drawn based on the experimental results and discussion.

1. The results demonstrate that the image analysis method can effectively describe the homogeneity of panels. From the image analysis results of the cut specimens of the wallboards, the commercial ceramsite concrete wallboard showed nonuniformity.
2. There was a serious nonuniform distribution of the aggregate of the commercial ceramsite concrete wallboard; the highest particle area increased from 18.9% at the bottom to 42.5% at the top, with a difference of approximately 24%.
3. From the bottom to the top of the wallboard, the density of ceramsite concrete specimens decreased from 1010 kg/m^3^ to 895 kg/m^3^, the corresponding compressive strength decreased from 10 MPa to 5 MPa, and the ultrasonic pulse velocity decreased from 2.5 km/s to 2.3 km/s.
4. According to experiments and regressions in this paper, the distribution of compressive strength, density, and ultrasonic pulse velocity had a linear relationship with the height of the specimens; they decreased with the increase in height from the specimens to the ground. They were negatively correlated with the proportion of ceramsite particle area. This result is dependent on the manufacturing process; different manufacturing processes may cause somewhat different results. In future research, more tests and analyses are needed to verify the same regular pattern for different manufacturing processes.

## Figures and Tables

**Table 1:** The proportions of the ceramsite concrete mixture (kg/m^3^).

Component	Proportion
Cement	750
Ceramsite	400
Filament	2
Foaming agent	0.5
Additive	0.7

## References

[1] Evola G., Marletta L. (2014). The effectiveness of PCM wallboards for the energy refurbishment of lightweight buildings. Energy Procedia.

[2] Liu P., Gong Y.F., Tian G.H., Miao Z.K. (2021). Preparation and experimental study on the thermal characteristics of lightweight prefabricated nano-silica aerogel foam concrete wallboards. Constr. Build. Mater..

[3] Kuznik F., Virgone J., Noel J. (2008). Optimization of a phase change material wallboard for building use. Appl. Therm. Eng..

[4] Han R., Qing Y. (2017). Study on the material performance of ceramsite concrete roof brick. Procedia Eng..

[5] Zhang Y.G., Shi Y.X., Shi J.B., Wang Q.X., Ni K., Zhang F.S. (2017). An experimental research on basic properties of ceramsite cellular concrete. Adv. Mater. Res..

[6] Fan L., Zhang Z., Yu Y., Li P., Cosgrove T. (2017). Effect of elevated curing temperature on ceramsite concrete performance. Constr. Build. Mater..

[7] Ji T., Zheng D.D., Chen X.F., Lin X.J., Wu H.C. (2015). Effect of prewetting degree of ceramsite on the early-age autogenous shrinkage of lightweight aggregate concrete. Constr. Build. Mater..

[8] Chen Y., Hui Q., Zhang H., Zhu Z., Wang C., Zhao J. (2020). Experiment and application of ceramsite concrete used to maintain roadway in coal mine. Meas. Control.

[9] Yew M.K., Yew M.C., Beh J.H., Saw L.H., Lim S.K. (2021). Effects of pre-treated on dura shell tenera shell for high strength lightweight concrete. J. Build. Eng..

[10] Dong J., Zhang T., Fu Y., Zheng B., Chai Y. (2019). Study on connection and properties of green assembled building steel structure. Results Phys..

[11] Shen Y., Liu B., Lv J., Shen M. (2019). Mechanical properties and resistance to acid corrosion of polymer concrete incorporating ceramsite, fly ash and glass fibers. Materials.

[12] Zhao H., Liu H., Wan Y., Ghantous R.M., Li J., Liu Y., Ni Y., Guan J. (2021). Mechanical properties and autogenous deformation behavior of early-age concrete containing pre-wetted ceramsite and CaO-based expansive agent. Constr. Build. Mater..

[13] Adhikary S.K., Ashish D.K., Rudžionis Ž. (2021). Aerogel based thermal insulating cementitious composites: A review. Energy Build..

[14] Xie J., Liu J., Liu F., Wang J., Huang P. (2019). Investigation of a new lightweight green concrete containing sludge ceramsite and recycled fine aggregates. J. Clean. Prod..

[15] Yang Y., Wu W., Fu S., Zhang H. (2020). Study of a novel ceramsite-based shape-stabilized composite phase change material (PCM) for energy conservation in buildings. Constr. Build. Mater..

[16] Sun Y., Li J., Chen Z., Xue Q., Sun Q., Zhou Y., Chen X., Liu L., Poon C.S. (2021). Production of lightweight aggregate ceramsite from red mud and municipal solid waste incineration bottom ash: Mechanism and optimization. Constr. Build. Mater..

[17] Zhuang Y.Z., Chen C.Y., Ji T. (2013). Effect of shale ceramsite type on the tensile creep of lightweight aggregate concrete. Constr. Build. Mater..

[18] Ahmad M.R., Chen B. (2019). Experimental research on the performance of lightweight concrete containing foam and expanded clay aggregate. Compos. Part B Eng..

[19] Adhikary S.K., Rudžionis Ž., Tučkutė S., Ashish D.K. (2021). Effects of carbon nanotubes on expanded glass and silica aerogel based lightweight concrete. Sci. Rep..

[20] Mi H., Yi L., Wu Q., Xia J., Zhang B. (2021). Preparation of high-strength ceramsite from red mud, fly ash, and bentonite. Ceram. Int..

[21] Li Y., Ding J. (2005). Segregation capability of fresh High Strength Lightweight Aggregate Concrete (HSLWAC). Concrete.

[22] Tenza-Abril A.J., Villacampa Y., Solak A.M., Baeza-Brotons F. (2018). Prediction and sensitivity analysis of compressive strength in segregated lightweight concrete based on artificial neural network using ultrasonic pulse velocity. Constr. Build. Mater..

[23] Jiang L., Lai X., Jiao H. (2020). Concrete relative velocity prediction to prevent mortar segregation for safe gravity transportation. Alex. Eng. J..

[24] Cano-Pleite E., Hernández-Jiménez F., Acosta-Iborra A., Tsuji T., Müller C. (2017). Segregation of equal-sized particles of different densities in a vertically vibrated fluidized bed. Powder Technol..

[25] Yan W., Cui W., Qi L. (2020). Effect of aggregate gradation and mortar rheology on static segregation of self-compacting concrete. Constr. Build. Mater..

[26] González-Taboada I., González-Fonteboa B., Pérez-Ordóñez J.L., Eiras-López J. (2017). Prediction of self-compacting recycled concrete mechanical properties using vibrated recycled concrete experience. Constr. Build. Mater..

[27] Yu L.L., Chen J.D., Wu Y.W. (2015). The performance of the ceramsite concrete block and its application research. Appl. Mech. Mater..

[28] Wang S., Ming Y., Han Z., Dong W., Fei L. (2013). Experimental Research on Crushed Ceramsite Concrete. Ind. Constr..

[29] Navarrete I., Lopez M. (2017). Understanding the relationship between the segregation of concrete and coarse aggregate density and size. Constr. Build. Mater..

[30] Huang H., Yuan Y., Zhang W., Liu B., Viani A., Macova P. (2019). Microstructure investigation of the interface between lightweight concrete and normal-weight concrete. Mater. Today Commun..

[31] German S., Brilakis I., DesRoches R. (2012). Rapid entropy-based detection and properties measurement of concrete spalling with machine vision for post-earthquake safety assessments. Adv. Eng. Inform..

[32] Zhou K., Lei D., He J., Zhang P., Bai P., Zhu F. (2021). Single micro-damage identification and evaluation in concrete using digital image correlation technology and wavelet analysis. Constr. Build. Mater..

[33] Lei M., Liu L., Shi C., Tan Y., Lin Y., Wang W. (2021). A novel tunnel-lining crack recognition system based on digital image technology. Tunn. Undergr. Space Technol..

[34] Sun Y., You J., Zhou J., Liu X., Yu L., Bu C., Yan Z., Chen X. (2020). Quantified research on the nonuniform distribution of expanded polystyrene beads in sandwich panels. Constr. Build. Mater..

[35] Wang G., Kong Y., Sun T., Shui Z. (2013). Effect of water–binder ratio and fly ash on the homogeneity of concrete. Constr. Build. Mater..

[36] Chen T.T., Wang W.C., Wang H.Y. (2020). Mechanical properties and ultrasonic velocity of lightweight aggregate concrete containing mineral powder materials. Constr. Build. Mater..

[37] Lu K., Xia D., Yang S. (2017). Experimental study on material m ix of new ceramic concrete. Concrete.

[38] Zhang Y., Liu B., Wu T., Chen X. (2018). Mechanical properties and microstructure of high-strength lightweight aggregate concrete with shale ceramist. J. Xi’an Univ. Archit. Technol. Nat. Sci. Ed..

[39] Ahmad M.R., Chen B., Shah S.F.A. (2019). Investigate the influence of expanded clay aggregate and silica fume on the properties of lightweight concrete. Constr. Build. Mater..

